# Supplementary material for: Pharmacokinetics and Tissue Distribution of Enavogliflozin in Mice and Rats
Source: Pharmaceutics. 2022 Jun 7;14(6):1210. doi: 10.3390/pharmaceutics14061210 (PMC9230590; doi:10.3390/pharmaceutics14061210)
Supplement: Supplementary file 1 [file pharmaceutics-14-01210-s001.zip › pharmaceutics-1730330-supplementary.pdf]

pharmaceutics

Supplementary File

Pharmacokinetics and tissue distribution of enavogliflozin in mice and rats

Minyeong Pang[1], So Yeon Jeon[1], Min-Koo Choi[1], Ji-Hyeon Jeon[2], Hye-Young Ji[3], Ji-Soo Choi[3], and Im-Sook Song[2,*]

The normal distribution of the data was assessed using the Shapiro-Wilk test for the pharmacokinetic parameters of enavogliflozin in both mice and rats following intravenous and oral administration of enavogliflozin (0.3, 1, and 3 mg/kg). SPSS for Windows sdftware (version 25.0; IBM Corp., Armonk, NY, USA) were used and a difference was considered significant at $p < 0.05$.

Citation: Pang, M.; Jeon, S.Y.; Choi, M.-K.; Jeon, J.-H.; Ji, H.-Y.; Choi, J.-S.; Song, I.-S. Pharmacokinetics and Tissue Distribution of Enavogliflozin in Mice and Rats. *Pharmaceutics* **2022**, *14*, 1210. https://doi.org/10.3390/pharmaceutics14061210

Academic Editor: José Martínez Lanao

Received: 1 May 2022
Accepted: 6 June 2022
Published: 7 June 2022

Publisher's Note: MDPI stays neutral with regard to jurisdictional claims in published maps and institutional affiliations.

Copyright: © 2022 by the authors. Licensee MDPI, Basel, Switzerland. This article is an open access article distributed under the terms and conditions of the Creative Commons Attribution (CC BY) license (https://creativecommons.org/licenses/by/4.0/).

Table S1. P values and Q-Q plot from the normality test for the pharmacokinetic parameters of enavogliflozin in mice following its intravenous administration using the Shapiro-Wilk test.

Parameters		Dose (mg/kg)		
		0.3	1	3
AUC_∞/D	P value	0.542	0.915	0.442
	Q-Q plot	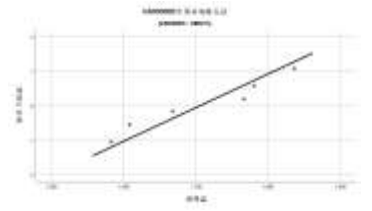	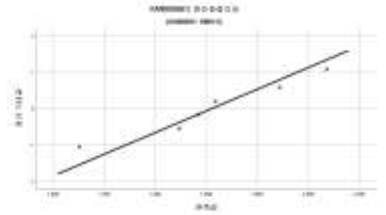	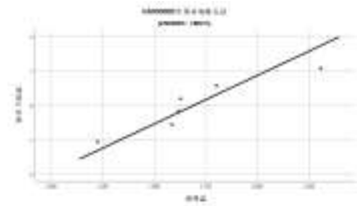
C_0/D	P value	0.874	0.098	0.645
	Q-Q plot	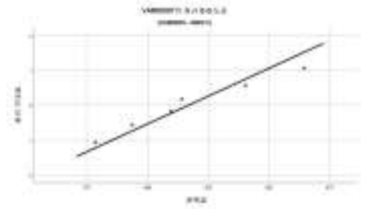	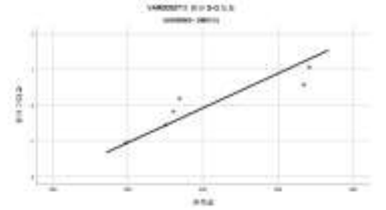	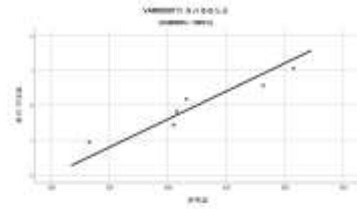
CL	P value	0.521	0.368	0.562
	Q-Q plot	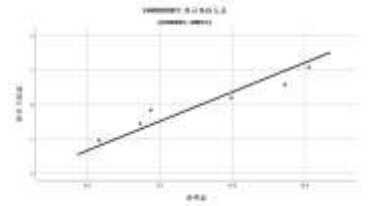	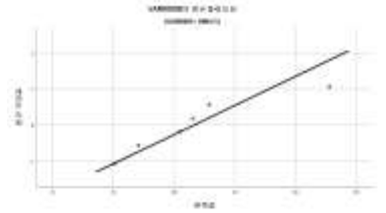	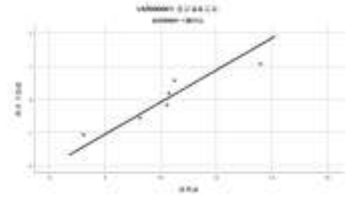
V_{dss}	P value	0.591	0.342	0.111
	Q-Q plot	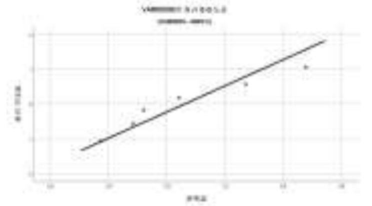	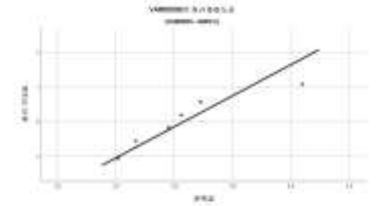	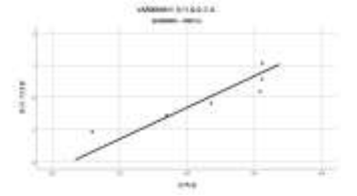
$T_{1/2}$	P value	0.843	0.587	0.454
	Q-Q plot	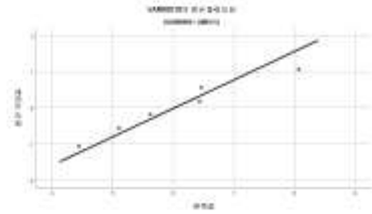	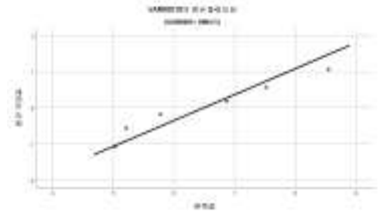	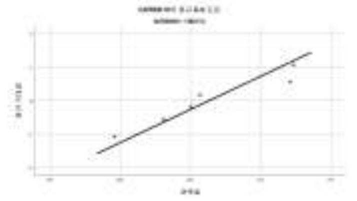
MRT	P value	0.913	0.524	0.807
	Q-Q plot	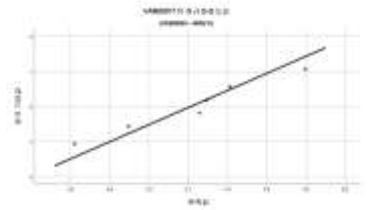	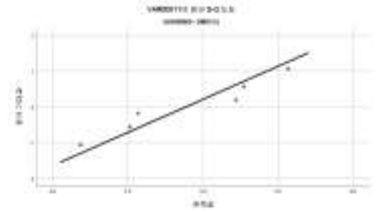	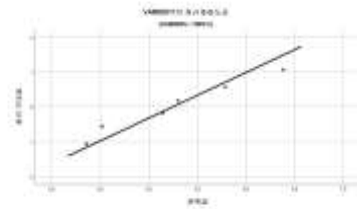

Table S2. P values and Q-Q plot from the normality test for the pharmacokinetic parameters of enavogliflozin in mice following its oral administration using the Shapiro-Wilk test.

Parameters		Dose (mg/kg)		
		0.3	1	3
AUC_∞/D	P value	0.405	0.809	0.758
	Q-Q plot	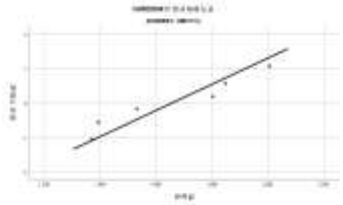	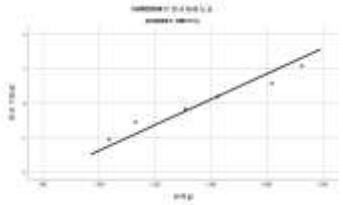	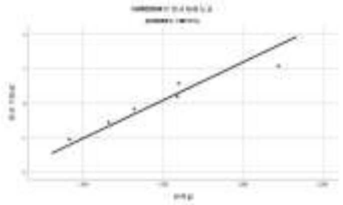
C_{max}/D	P value	0.455	0.292	0.982
	Q-Q plot	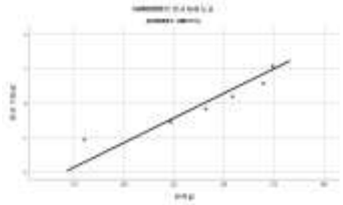	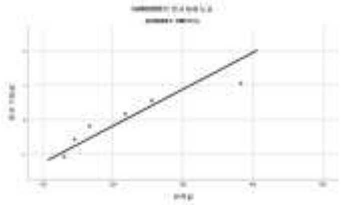	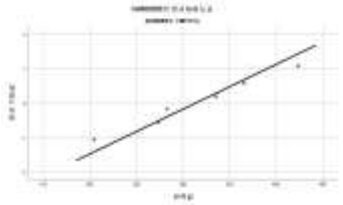
T_{max}	P value	0.184	0.197	0.081
	Q-Q plot	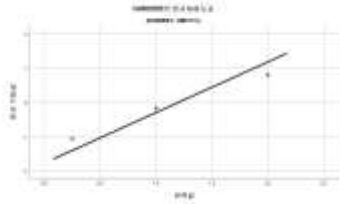	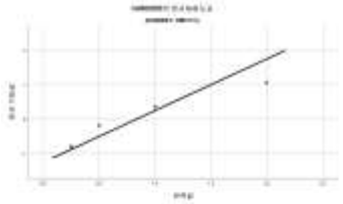	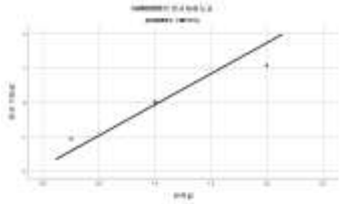
$T_{1/2}$	P value	0.456	0.139	0.79
	Q-Q plot	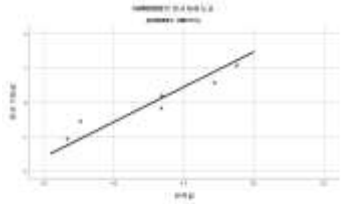	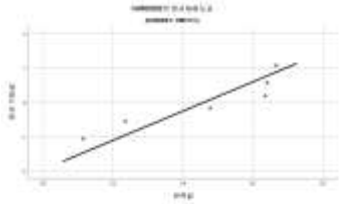	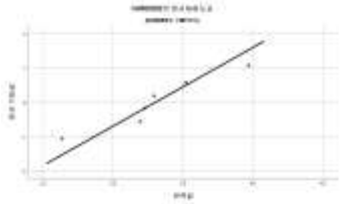
MRT	P value	0.876	0.423	0.196
	Q-Q plot	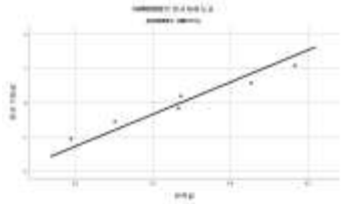	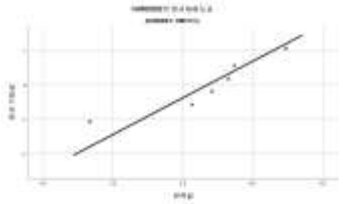	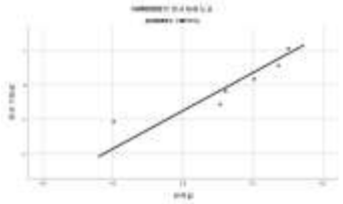

Table S3. P values and Q-Q plot from the normality test for the pharmacokinetic parameters of enavogliflozin in rats following its intravenous administration using the Shapiro-Wilk test.

Parameters		Dose (mg/kg)		
		0.3	1	3
AUC_{∞}/D	P value	0.278	0.704	0.315
	Q-Q plot	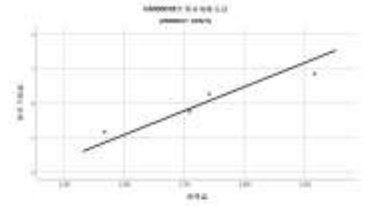	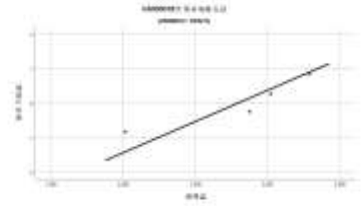	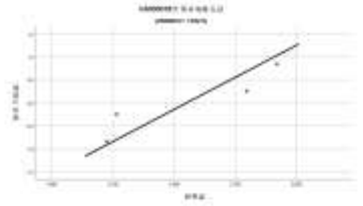
C_0/D	P value	0.280	0.105	0.808
	Q-Q plot	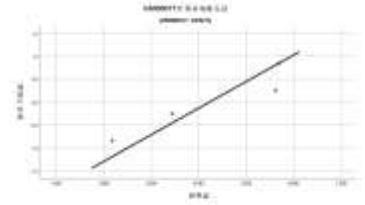	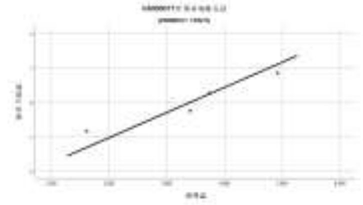	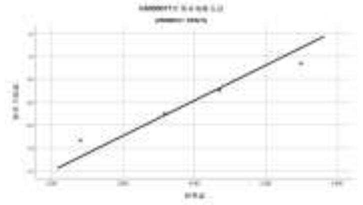
CL	P value	0.891	0.133	0.156
	Q-Q plot	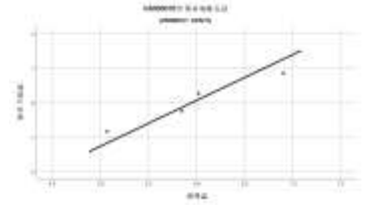	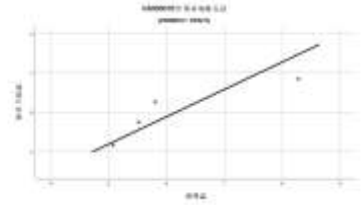	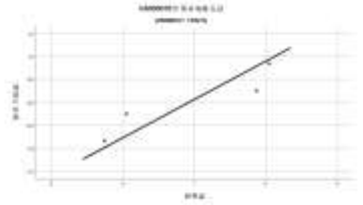
V_{dss}	P value	0.559	0.065	0.524
	Q-Q plot	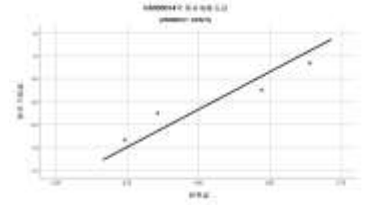	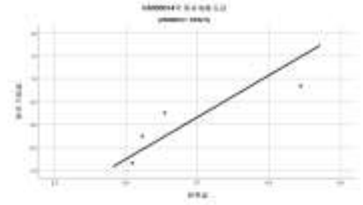	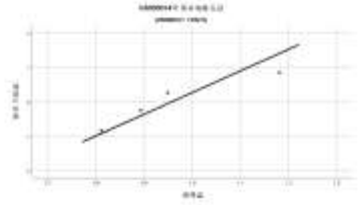
$T_{1/2}$	P value	0.278	0.704	0.315
	Q-Q plot	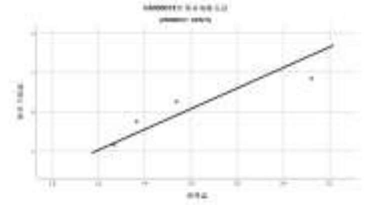	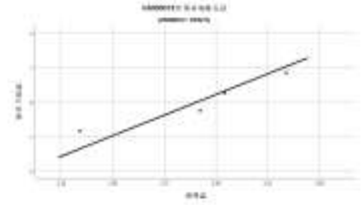	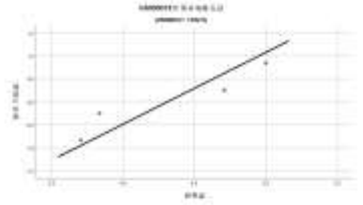
MRT	P value	0.663	0.206	0.976
	Q-Q plot	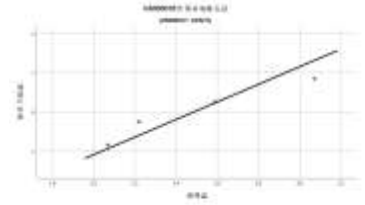	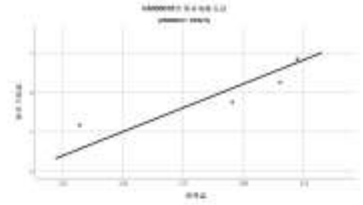	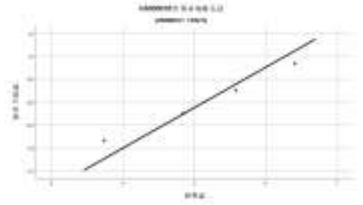

Table S4. P values and Q-Q plot from the normality test for the pharmacokinetic parameters of enavogliflozin in rats following its oral administration using the Shapiro-Wilk test.

Parameters		Dose (mg/kg)		
		0.3	1	3
AUC_{∞}/D	P value	0.132	0.606	0.580
	Q-Q plot	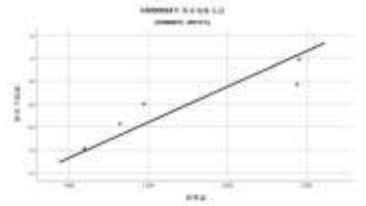	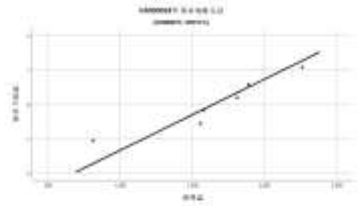	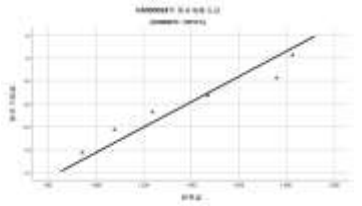
C_{max}/D	P value	0.471	0.124	0.887
	Q-Q plot	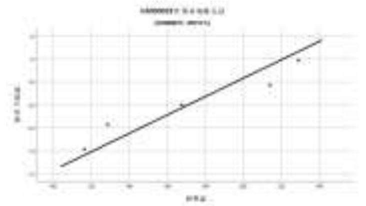	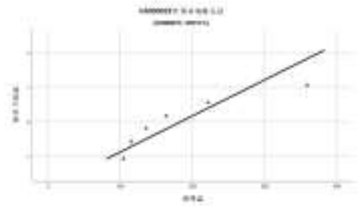	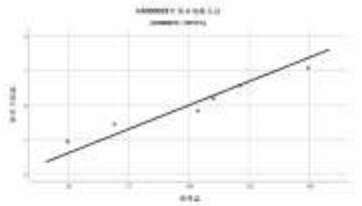
T_{max}	P value	0.149	0.070	0.613
	Q-Q plot	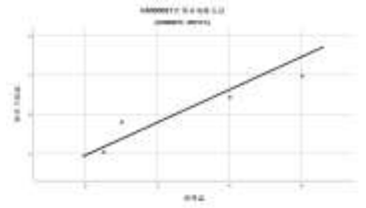	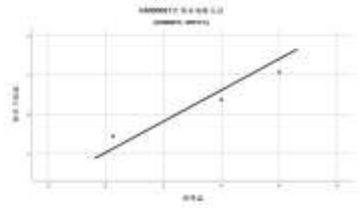	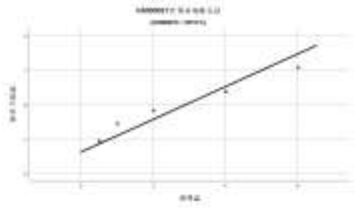
$T_{1/2}$	P value	0.261	0.455	0.053
	Q-Q plot	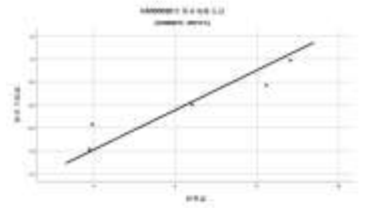	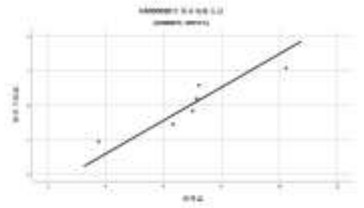	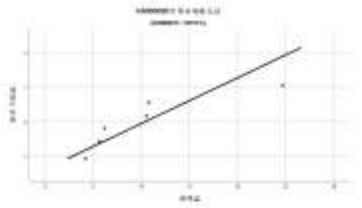
MRT	P value	0.731	0.178	0.099
	Q-Q plot	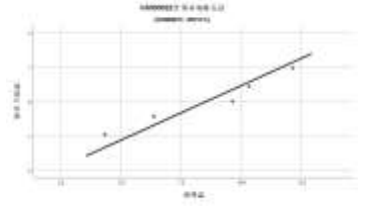	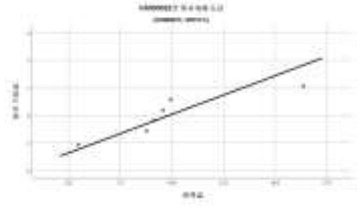	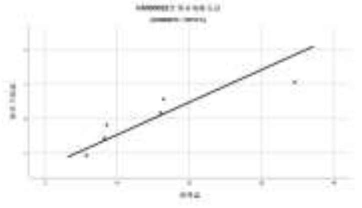

Table S5. P values and Q-Q plot from the normality test for AUC values and AUC ratios of enavogliflozin in various tissues after single or repeated oral doses (1 mg/kg) of enavogliflozin in mice using the Shapiro-Wilk test.

Tissue		AUC_{48h} (µg·h/mL for plasma or µg·h/g tissue)		
		Single dose	Repeated dose for 7 days	Repeated dose for 14 days
Plasma	P value	0.644	0.938	0.165
	Q-Q plot	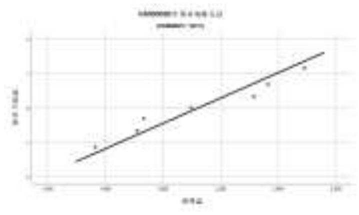	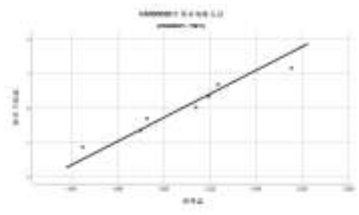	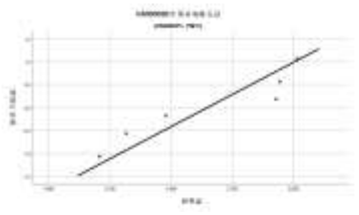
Kidney	P value	0.966	0.151	0.453
	Q-Q plot	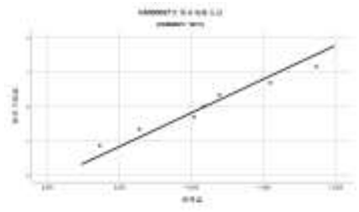	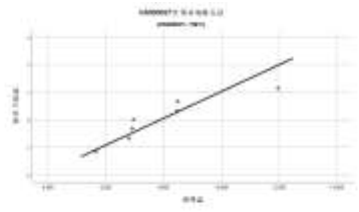	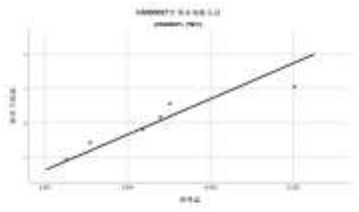
Liver	P value	0.123	0.199	0.711
	Q-Q plot	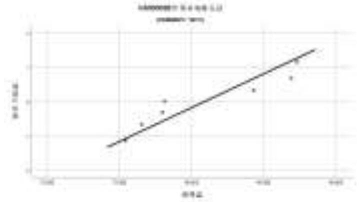	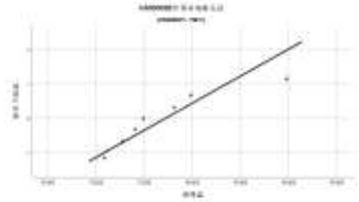	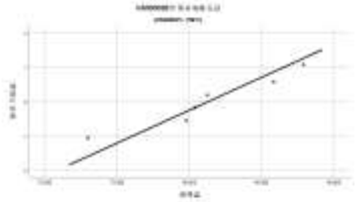
Small intestine	P value	0.410	0.458	0.078
	Q-Q plot	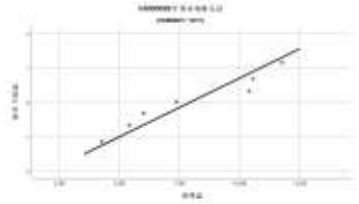	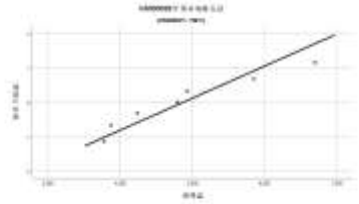	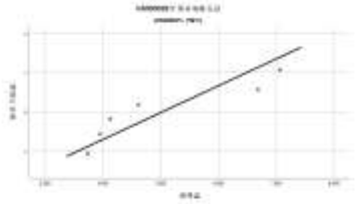
Large intestine	P value	0.746	0.244	0.191
	Q-Q plot	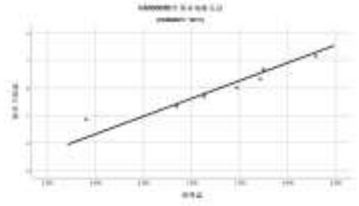	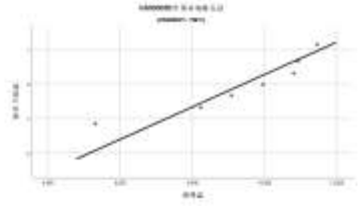	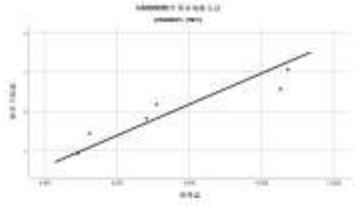
Tissue		AUC ratio		
Kidney	P value	0.618	0.446	0.646
	Q-Q plot	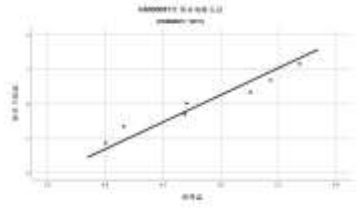	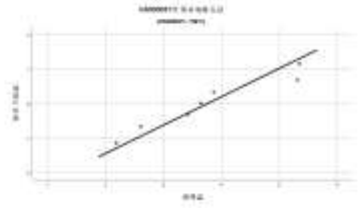	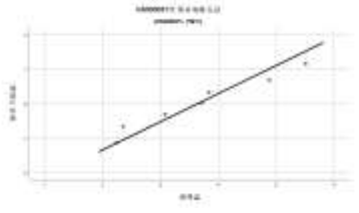

Liver	P value<br>Q-Q plot	0.883<br>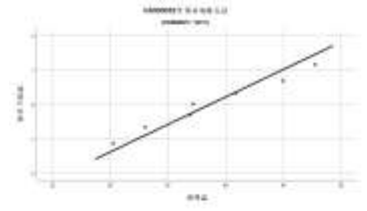	0.623<br>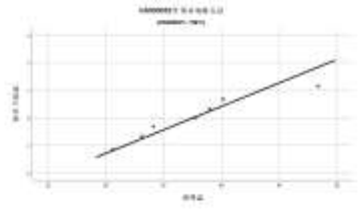	0.134<br>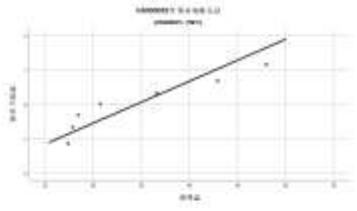
Small intestine	P value<br>Q-Q plot	0.264<br>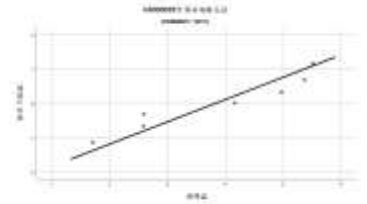	0.055<br>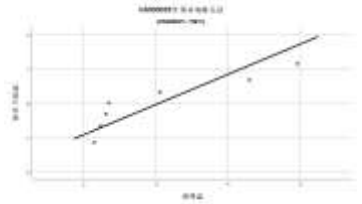	0.164<br>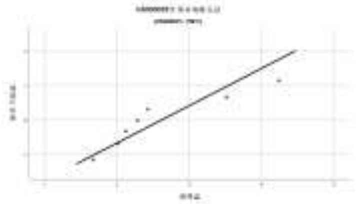
Large intestine	P value<br>Q-Q plot	0.320<br>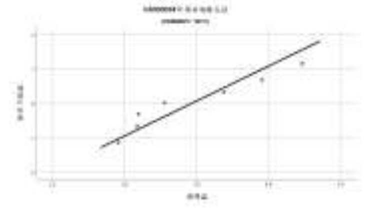	0.138<br>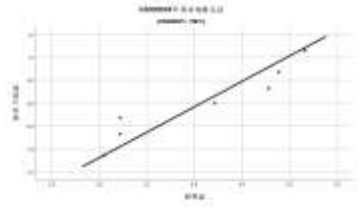	0.437<br>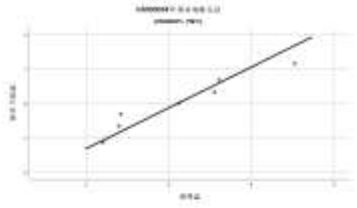
